# New integrated hydrologic approach for the assessment of rivers environmental flows into the Urmia Lake

**DOI:** 10.1038/s41598-022-10262-4

**Published:** 2022-05-16

**Authors:** Ali Mobadersani, Ali Hosseinzadeh Dalir, Mehdi Yasi, Hadi Arvanaghi, Mark J. Kennard

**Affiliations:** 1 Department of Water Science and Engineering, University of Tabriz, Tabriz, Iran; 2 Department of Irrigation and Reclamation Engineering, University of Tehran, Karaj Campus, Karaj, Iran; 3 Australian Rivers Institute, Griffith University, Nathan, Qld Australia

**Keywords:** Hydrology, Environmental impact, Wetlands ecology

## Abstract

Recent research has greatly focused on the environmental water supplement of rivers individually and independently. However, a comprehensive and integrated view of all rivers in the basin is simultaneously required in closed basins leading to lakes and wetlands. This has affected Lake Urmia, which is the second largest saltwater lake in the world. It has been in danger of drying up in recent years as a result of not allocating the required environmental flow (e-flow) due to the increase in water resource consumption in the agricultural sector and climate changes. In this study, a method derived from the flow duration curve shifting (FDCS) method is presented in addition to explaining the possibility of providing the e-flow of rivers leading to the lake. The method can make the least amount of change in the hydrological characteristics of rivers while providing the volume of required water by the ecosystem of lakes or downstream wetlands. Unlike the conventional method which presents the results on a monthly basis, the above-mentioned method is based on daily data of hydrometric stations and can calculate the amount of the environmental requirement of rivers in real-time according to the upstream inlet of the river. This method has been used in the Urmia Lake basin. According to the results, it can provide the environmental requirement of the lake by allocating 70.5% of the annual flow of rivers and thus can save the lake and the ecosystem of the region from the current critical conditions.

## Introduction

Nowadays, intense competition between ecosystem and water resource development projects, especially in rivers has emerged with the increasing population and the human need for water^1^. Many aquatic ecosystems are declining and disappearing due to human interventions in the overuse of water resources^2,3^ and climate change^4–6^. Environmental flow (e-flow) allocation is an important factor in maintaining ecosystems, along with flora and fauna species^7^. Given the importance of this type of flow and for the convergence of the applied concepts in these studies in Brisbane declaration^8^, the quantity, time, and quality of the required water flow for the sustainability of river ecosystems and estuaries^9^ were known as e-flow. This definition emphasizes the maintenance of the regime of natural river flows^10,11^.

In recent decades, more than 200 methods have been proposed for estimating e-flows that are mainly based on hydrological time series due to their ease of use^12^. In addition, some of the proposed methods (e.g., Tennant^13^) have a simple basis while other ones (e.g., holistic methods^14,15^) have studied the general dimensions of the ecosystem and social conditions of the region^16,17^. The complexity of estimating e-flows in these methods has increased due to the raise in the number of parameters and the use of multi-objective optimization algorithms^18,19^. An echohydrological method (combination of ecological and hydrological methods) is used to increase the efficiency and compatibility of hydrological methods with the ecosystem of each region. In this regard, selecting the adequate ecosystem conservation criteria^20–22^ in allocating e-flows^23,24^ is one of the most important challenges in estimating the appropriate e-flow for a basin in ecohydrological methods^25^. Thus, hydraulic methods^26^ have been employed in some studies to determine the class of hydrological environmental management and e-flow allocation^27,28^. Other studies emphasize methods based on water quality^29,30^ while considering the importance of the spatial changes of qualitative and hydrological parameters in the performance of e-flow in the restoration of river ecosystems^31^. However, the water supply adequacy of downstream wetlands has been mentioned as an ecological criterion for e-flow allocation in another research^32^.

According to global studies, a decade after the first declaration^8^, the supply of the required water for aquatic ecosystems has received more attention^33,34^. Considering the needs of these ecosystems such as lakes and wetlands^35^, in the second statement, water level parameters were added to the original definition to comprehend the definition of e-flow^36^. Based on this explanation, previous methods should be reviewed to provide downstream ecosystem water^37^, and the comprehensive method should be introduced accordingly. It is noteworthy that only the water need of rivers leading to the wetland has been estimated in some studies^38^. In another study, the water requirement of wetlands and lakes without the e-flow of rivers has been considered and determined by the annual flow (AF) volume to reach a certain level^39^. Despite all presented studies, it should be mentioned that each river, wetland, or lake has been independently in these methods. If the rivers are in an Endorheic basin leading to a lake or wetland, the ecosystem of the area under their coverage must be rehabilitated, and the environmental needs of the lake ecosystem should be assessed collectively. To study this type of river e-flow, it is necessary for all rivers to restore the downstream lake or wetland using an integrated method, and each river must be evaluated separately.

Ecosystems in different basins of Iran are not in a good condition due to excessive use of water resources^40^, and this issue is tangible in some water basins such as the Urmia Lake^41,42^. Increased water withdrawals from agriculture^43^, households, and industry, on the one hand, and climate change, on the other, have led to excessive declines in water levels. As a result, less than 10% of the lake has remained in comparison with its optimal conditions. The sharp decline in lake water levels has been associated with reduced tourism activity and damage to agriculture in the region, having a detrimental effect on urban and rural infrastructure and the livelihood of families in this area^44,45^. The salinity of the lake water increased by a decrease in the lake water volume. Therefore, extinction threatens the dominant species of the lake and causes major problems in the food chain of this ecosystem^46,47^. According to the Urmia Lake Restoration Program, there are ongoing government efforts to improve the condition of the lake. Although the lake has reached a relative stabilization through precipitations in recent years and thanks to the taken measures, the current condition of the lake is far from its ecological level^48^. In this research, an integrated ecohydrological method was utilized to estimate the e-flow requirement (EFR) of rivers leading to the lake and maintain the environmental water requirement (EWR) of the lake. This study was performed on the basis of Urmia Lake and its supplying rivers. The environmental conditions of rivers and lakes were independently examined based on previous studies, followed by presenting a suitable integrated method for allocating the required e-flow for each river leading to the lake.

## Materials and methods

### Specifications of the study area

Urmia Lake, as the largest inland lake of Iran, is a national park and one of the largest Ramsar sites of Iran (Ramsar, 1971). The lake is formed in a natural depression within the catchment area in the northwest of Iran. The basin of the lake covers an area of 52,000 km^2^ and its area is about 5,700 km^2^^49^. In addition, its maximum length and width are 140 and 50 km, respectively. Further, the lake catchment is a closed inland basin in which all rainwater runoff flows to the central saline lake, and evaporation from the surface of the lake is the only way out. More importantly, it is the largest saltwater lake in Iran and the second largest saltwater lake in the world.

The current surface flow system to Urmia Lake consists of 10 main rivers with permanent flow potential, including Zola, Nazlu, Rozeh, Shahrchai, Baranduz, Gadar, Mahabad, Simineh, Zarrineh, and Aji. In terms of the water supply potential of Urmia Lake, Zarrineh, Simineh, Aji, and Nazlu rivers with a flow allocation of 41, 11, 10, and 6% have a key role, respectively.

**Figure 1:**
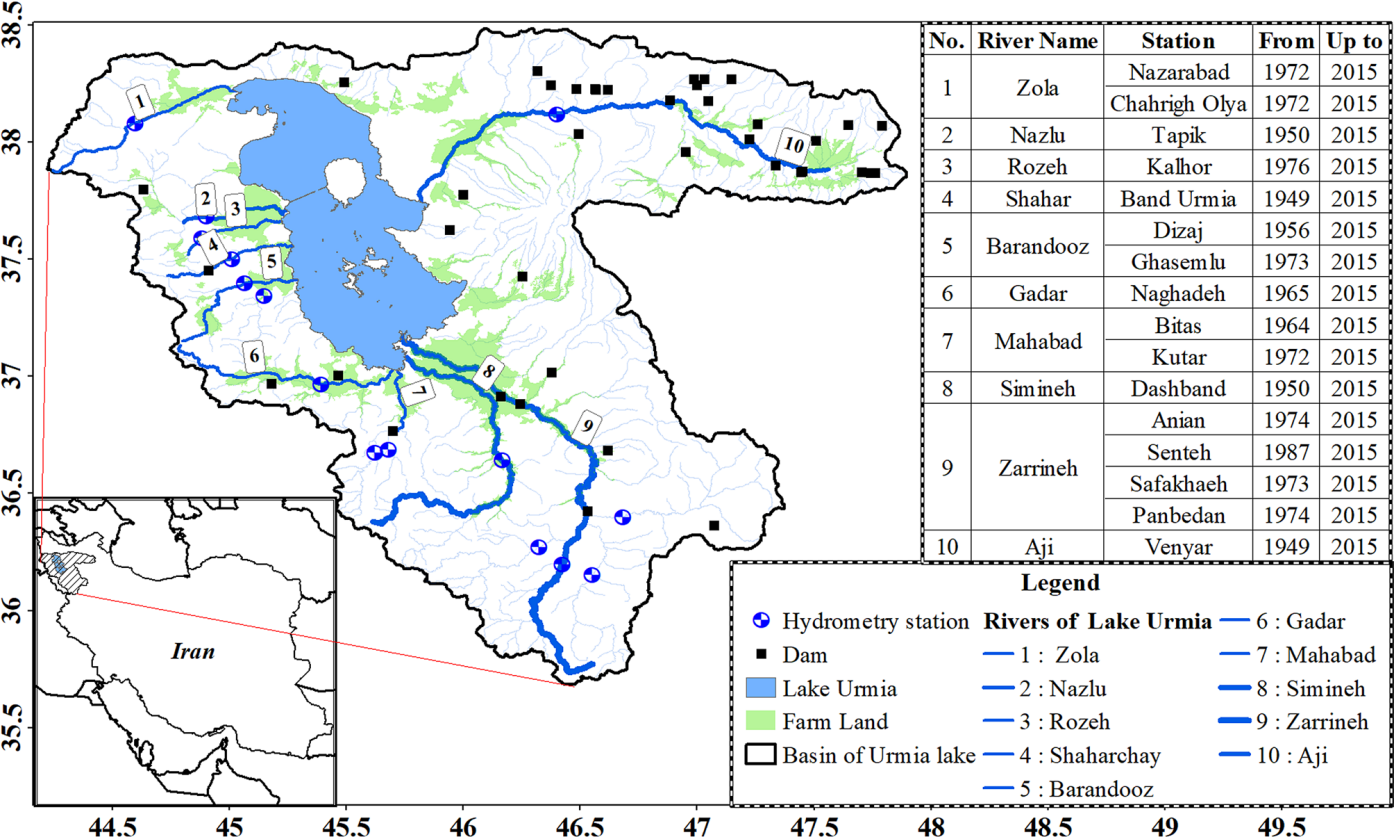
An overview of the Urmia Lake basin, the rivers, and selected gauging stations. Figure 1 was generated by ArcGIS v10.2 software^50^ (Environmental Systems Research Institute, Inc., USA, URL http://www.esri.com/).

The rivers of this basin are originated from mountains and pass through the heights and enter the agricultural plains. The main usage in plains are for agriculture which cause the changes in natural rivers flow regime. On the other hand, the natural flow regime of the rivers should be considered as the basis for e-flow calculation. So, in the current study the obtained data from the stations situated in the upstream of the rivers and the stations before the agricultural plains are utilized to alleviate the effects of agricultural use on natural flow regime of the rivers. Also, to eliminate the effects of dam rule curve on river flow regime, stations situated in the upstream of the dams are considered as the main scale in the upstream of the dammed rivers like Zarrineh, Mahabad and Zola. Despite all the efforts made to select stations with the least human impact, the two stations related to Aji and Shahar Rivers have been affected by the structures built above them. Therefore, in order to eliminate the effects of the constructed structures at the upstream of the stations, flow naturalization methods were used only for the two stations of Venyar of Aji River and the Band Urmia station of Shahar River. There are several ways to naturalize hydrometric station data. Terrier et al.^51^ by studying flow naturalization methods in various researches were able to provide a comprehensive study of naturalization methods and selection criteria for each of these methods. According to their studies, the first and the most important prerequisite for stream naturalization is to identify the factors affecting the river and the quality of data in the region, which play a major role in choosing the flow naturalization method. Two factors play a major role in affecting river hydrology. The first factor is the construction of hydraulic structures along the path of rivers and the second factor is the change of land use that has occurred in the rivers basin. In the current study, the purpose of flow naturalization is to eliminate the effects of large dams built on the inlet rivers of the lake, which can affect the hydrology of the river flow. It should be noted that it is not possible to eliminate the effects of land use change due to the gradual nature of the changes, the inability to determine the exact amount and time of the changes and the lack of required data as well. Therefore, in this study, the effects of land use change at the upstream of the stations have been neglected. The most important reason that the Aji River needs to naturalize is the existence of several small dams upstream of Venyar station. To eliminate the effects of dams and flow naturalization at the upstream of this station, the spatial interpolation method introduced by Hughes and Smakhtin^52^ was used. In this method, Sahzab hydrometric station located at the upstream of the river was used as a base station to naturalize the flow. The next station which needs to be naturalized the flow is the Band Urmia station Shahar River. The main problem for this river has been the construction of a dam upstream of Band Urmia river station since 2004. The drainage area ratio method introduced by Hirsch^53^ was used to eliminate the effect of this dam on the station data. This method has been used by various researchers to naturalize river flow^54–56^ which is based on the upstream drainage area of the stations. In this method the ratio of the drainage area of the two stations is used to naturalize the flow in the affected station. For this purpose, the data of Bardehsoor station located upstream of the dam was used to naturalize the data of the Band Urmia station. So, anthropogenic effects are at the minimum level in calculations. The utilized stations to calculate the e-flow as upstream stations are illustrated in Fig. 1.

### Appropriate criteria for allocating the EWR of the Urmia Lake

Due to the high salinity of Urmia Lake, only a small number of invertebrates make up the living organisms of this huge water body. Saltwater shrimp or Artemia is a type of aquatic crustacean which can be found in saltwater lakes or coastal lagoons worldwide. Artemia can tolerate salinity less than 10 gl^−1^ up to 340 gl^−1^ and adapt to environmental conditions. Artemia Urmiana, the most well-known species of the Urmia Lake, is considered as the main food of migratory birds that spend part of their wintering period on the lake and surrounding wetlands. The presence of this species in the Urmia Lake was first reported by Gunter (1899), and many researchers have confirmed the existence of this bisexual creature in this lake^57–61^.

One of the key factors in estimating the EWR of Urmia Lake is to create an appropriate environmental condition for its dominant species. Abbaspour and Nazari Doost^39^ identified the EWR of the Urmia Lake by considering the living conditions of Artemia as its dominant species. In this study, Artemia Urmaina was selected as a biological indicator, along with NaCl and elevation above mean sea level (AMSL) as the indicators of water quality and quantity, respectively. The combination of these three indicators forms the ecological basis of Urmia Lake. Therefore, salinity is considered to be equal to 240 gl^−1^ as the tolerable limit of the biological index. Using long-term statistics in the Urmia Lake and the relationship between quantitative and qualitative water indicators, the water level of 1274.1 m (AMSL) was chosen as the ecological level of the lake so that the balance of these three indicators remained within the allowable range. The study indicated that the calculated environmental water demand of Urmia Lake was equal to 3084 Mm^3^ per year provided by main rivers entering the lake. Therefore, the proposed new methods should be able to deliver this volume of water to the lake and simultaneously feed the EFR of the river. To supply this water volume, government has programs in order to mitigate the water consumption especially in agriculture. The most important program is 40 percent reduction in agricultural water consumption which is accompanied with the increase of efficiency. Also the government pursues urban wastewater treatment to retrieve some of domestic water to the lake. The mentioned programs are time consuming, however, the new methods presented in this study can be useful for managers in determining the allocation patterns and consumption management.

###  Ordinary method of flow duration curve shifting (FDCS) in estimating e-flow

Since the early 1990s, various methods have been developed based on the hydrological indices^62^ in order to determine the e-flow by taking into account the flow variability and adaptation to the ecological conditions of rivers. One of the intended diagrams in the study of the hydrological characteristics is the flow duration curve (FDC), which is used to assess the fluctuations and variability of water flow from an environmental point of view. Given the importance of the presence of flood currents in the restoration of the river and wetland ecosystems^63,64^, the FDC is one of the most practical methods to show the full range of river discharge characteristics from water shortage to flood events. This diagram also demonstrates the relationship between the amount and frequency of the flow which can be prepared for daily, annual, and monthly time intervals^65^. The FDCS is a method in which FDC is employed to estimate the river flow. This method was introduced by Smakhtin and Anputhas^66^ to evaluate the e-flow in the river system. The method, which is called FDCS, provides a hydrological regime to protect the river in the desired ecological conditions.

In the previous research, most of the rivers in the Urmia Lake basin have been compatible with FDCS, and due to the lack of biological data regarding these rivers, it is always one of the top priorities among the methods of estimating the e-flow in rivers leading to the Urmia Lake^67,68^. It is noteworthy that the characteristics of calculation steps of the ordinary method are provided as follows.

This method consists of four main steps:

1. Assessing the existing hydrological conditions (preparing the FDC for a natural river flow regime),
2. Selecting the appropriate environmental management class;
3. Acquiring the environmental FDC;
4. Generating e-flow time series.

The first step is to prepare the FDC in the desired river range using monthly flow data. In this method, FDC for the natural river flow regime is prepared by 17 fixed percentage points of occurrence probabilities (0.01, 0.1, 1, 5, 10, 20, 30, 40, 50, 60, 70, 80, 90, 95, 99, 99.9, 99.99) where P_1_ = 99.99% and P_17_ = 0.01% represent the highest and lowest probability of occurrence, respectively. These points ensure that the entire flow range is adequately covered, as well as facilitating the continuation of the next steps.

This method, which uses mean monthly flow (MMF) data, considers six environmental management classes (EMC) from A to F. The FDC of EFR (FDC-EFR) for each class in terms of EMC is determined based on the obtained natural river FDC by the MMF. The higher EMC needs more water to maintain the ecosystem. These classes are determined based on empirical relationships between the flow and ecological status of rivers, which currently have no specific criteria for identifying these limits. The selection of the appropriate class individually relies on the expert’s judgment of the river ecosystem condition.

After obtaining the natural FDC, the next step is to calculate the FDC-EFR for each EMC using the lateral shifts of FDC to the left along the probabilistic axis. For EMC-A rivers, one lateral shift to the left is applied while two, three, and four lateral shifts are employed for EMC-B, EMC-C, and EMC-D rivers, respectively. It should be noted that the overall hydrological pattern of the flow will be maintained although the flow variation is lost for each shift.

In the current study, global e-flow calculation (GEFC) v2.0 software^69,70^ has been utilized to compute the e-flow by the FDCS method. The long-term data (at least 20 years) of MMF are the required input data for this software.

According to the research conducted on the rivers of the Urmia Lake basin, EMC-C is the minimum considered EMC for 10 main rivers of the lake, thus the EMC-C has been considered in this study, and all calculations for classes A, B, C have been performed accordingly.

### The description of new methods based on ordinary method

The main purpose of presenting new methods is to combine the EWR of wetlands or lakes and the hydrological method of FDCS, which can be used to calculate the e-flow of rivers and meet the needs of lakes or wetlands in downstream. These methods relies on the FDCS while with the difference that the proposed method includes three fundamental changes compared to the original one.

1. Applying monthly FDC (FDC for each month separately) instead of annual FDC,
2. Employing daily flow data instead of MMF,
3. Considering the downstream EWR in the amount of the lateral shift in the FDCS method.

The use of the structure of new methods lead to a dynamic process that is based on the selected EMC of the river, the amount of the natural flow, and the date of occurrence and can compute the amount of the e-flow of the river on each day of the year.

**Figure 2:**
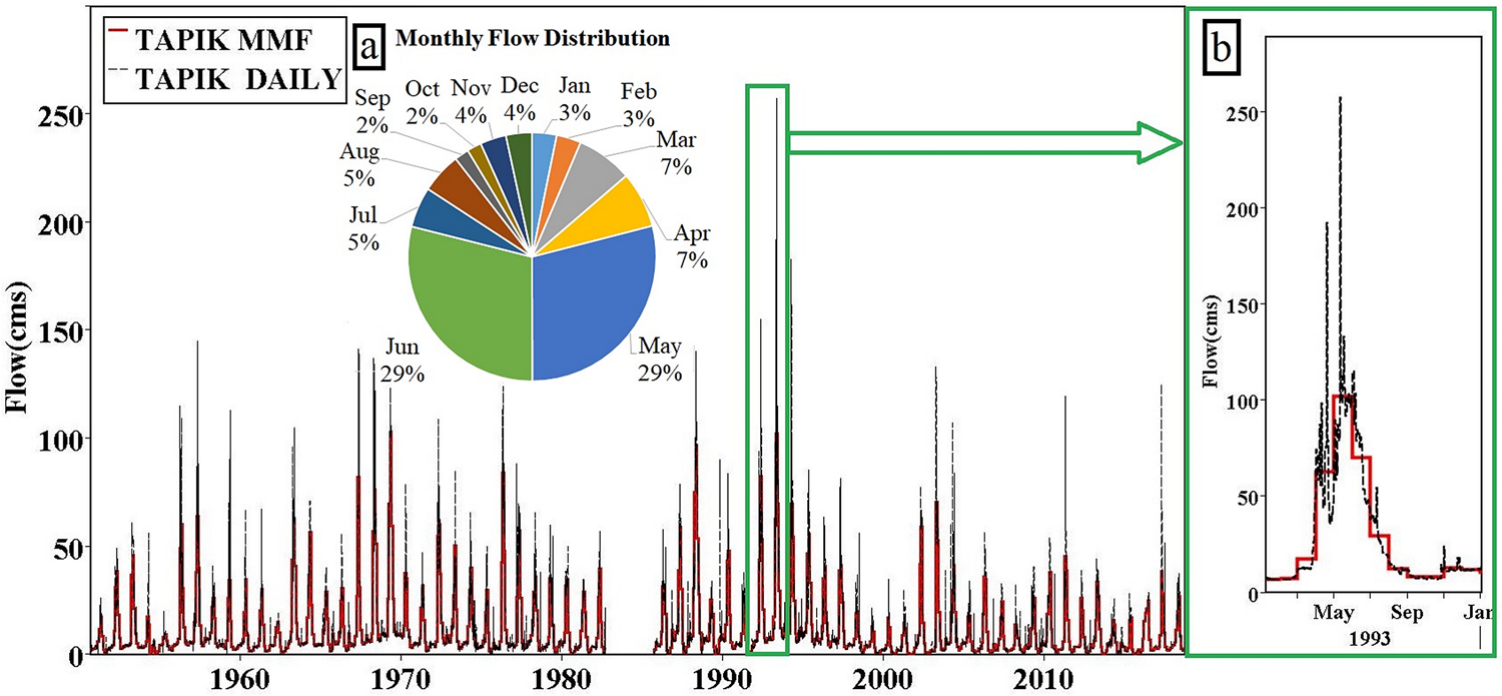
Historical hydrograph at the Tapik Station, Nazlu River: (**a**) Daily and mean monthly distribution of flows and (**b**) Magnified hydrograph for a typical year (1993).

River hydrology greatly varies depending on the type of the basin, the climate of the area, and the relationship between the basin and the river each exhibiting different behaviors during the months of the year. Accordingly, the proposed methods should provide sufficient comprehensiveness in estimating the e-flow by considering different flow characteristics. Due to the type and timing of precipitation in the Urmia Lake basin, the rivers are full of water from March to June and spend extremely less flows during the other times of the year. For example, Fig. 2 shows the distribution of the Nazlu River flows in the west of Urmia Lake throughout the year. According to the data, 74% of the AF crosses the river from March to June, and the highest and lowest river discharges are related to May with 29% and September and August with 2% of the AF, respectively.

According to the flow distribution throughout the year, the annual FDC is an average FDC of each month of the year. However, the flow of a river during the months of the year represents significant changes. Therefore, the monthly FDC is higher than the annual FDC in the high-water months (e.g., May). Additionally, this curve is lower compared to the annual FDC in the low-water months (e.g., September). Accordingly, the use of monthly FDCs provides more details of changes in the hydrological parameters of the flow and can be a better indicator of the hydrological index of river flows.

In the conventional FDCS method, the FDC is obtained using the MMF data of each station. The obtained curve represents the monthly average of river flow and does not illustrates the minimum, maximum and the effect of flow fluctuations in the estimation of e-flows (Fig. 2).

In the new methods, all FDC diagrams were obtained by daily data. Both annual (EFR-Ann) and monthly (EFR-Mon) methods are separately utilized to compare the calculation of the e-flow and to choose the best method. The annual FDC is a probabilistic chart for the whole year and the monthly FDC includes 12 probability curves for each year. Due to the use of FDC in e-flow estimations, it has been attempted to perform all calculations from this diagram. Therefore, concepts related to the flow volume can be integrated with the FDCS method. Some of the applied concepts for this purpose are as follows.

In the FDCS method, the FDC is defined based on 17 probabilistic percentage points. To calculate the mean AF (MAF) volume, the theorem of the mean value for a definite integral is employed in the FDC diagram. Accordingly, considering that FDC is continuous between the first and seventeenth probability points, the mean flow (*F*_*m*_) is obtained from Eq. (1) as.

1 $$F_{m} = \frac{1}{{P_{1} - P_{17} }}\mathop \smallint \limits_{{P_{17} }}^{{p_{1} }} F\left( p \right)dp$$

*F*_*m*_ = Mean flow. *P*_1_, *P*_*17*_ = Points of FDC probability that *P*_*1*_ = 99.99 and *P*_*17*_ = 0.01.

Given that the FDC consists of 17 probability points and the probability function ‘*F*(*P*)’ is unavailable for this curve as a mathematical equation, obtaining this equation for each flow curve increases the computational cost. Therefore, numerical integration methods can be used in this regard. The trapezoidal numerical solution method has been utilized for this purpose. By applying the trapezoidal method in solving Eq. (1), Eq. (2) is obtained, which is used to compute the mean flow of the FDC.

2 $$F_{m} = \frac{1}{{P_{1} - P_{17} }}\mathop \sum \limits_{i = 1}^{17} \frac{{\left( {F_{i} + F_{i + 1} } \right)}}{2}{*}\left[ {P_{i} - P_{i + 1} } \right]$$

*P*_*i*_ = 17 points of FDC probability that *P*_*1*_ = 99.99% and *P*_*17*_ = 0.01%. *F*_*i*_ = The amount of the river flow with the probability of the occurrence of *P*_*i*_*.*

To calculate the AF volume by monthly and annual FDCs, Eq. (3) can be applied for the AF volume in the EFR-Ann method, as well as employing Eqs. (4) and (5) for the monthly and AF volume in the EFR-Mon method, respectively.

3 $${\text{V}}_{{AF_{Ann} }} = \frac{365*24*3600}{{P_{1} - P_{17} }}\mathop \sum \limits_{i = 1}^{17} \frac{{\left( {F_{i} + F_{i + 1} } \right)}}{2}{*}\left[ {P_{i} - P_{i + 1} } \right]$$

4 $${\text{V}}_{Monthly } = \frac{{D_{k} *24*3600}}{{P_{1} - P_{17} }}\mathop \sum \limits_{i = 1}^{17} \frac{{\left( {F_{i} + F_{i + 1} } \right)}}{2}{*}\left[ {P_{i} - P_{i + 1} } \right]$$

5 $${\text{V}}_{{AF_{Mon} }} = \mathop \sum \limits_{k = 1}^{12} \left[ {{\text{V}}_{Monthly } } \right]_{k}$$

V_*AFAnn*_ = AF volume using annual FDC. V_*Monthly*_ = Monthly flow volume. V_*AFMon*_ = AF volume using monthly FDC. *D*_*k*_ = Number of the days of the *k*th month. *k* = Number of each month.

The required e-flow by wetlands and lakes must have two basic characteristics. The volume of EWR for maintaining their ecological level must be determined and provided by the studies of their ecosystems. In addition, fluctuations must be maintained in water levels in the lake due to hydrological conditions under the basins of the lake supplying rivers given the fact that maintaining the hydrological conditions of the river is one of the major goals of the FDCS method in estimating the e-flow of the river. On the other hand, the rehabilitation of the wetland or lake downstream of rivers requires a certain amount of water, and the new methods must be applied to combine these two goals. In this regard, the AF volume, which can be transferred to the lake (V_*L Mon or Ann*_) by these rivers, is calculated by taking into account the natural flow conditions of the rivers in the basin and without considering the consumptions,.

6 $${\text{V}}_{{L_{Ann} }} { } = \mathop \sum \limits_{j = 1}^{{\text{n}}} \left[ {{\text{V}}_{{AF_{Ann} }} } \right]_{j}$$

7 $${\text{V}}_{{L _{Mon} }} = \mathop \sum \limits_{j = 1}^{{\text{n}}} \left[ {{\text{V}}_{{AF_{Mon} }} } \right]_{j}$$

n = Number of input rivers to the lake. V_*LAnn*_ = AF volume, which can be transferred to the lake using annual FDC. V_*LMon*_ = AF volume, which can be transferred to the lake using monthly FDC.

The ratio of the EWR of the lake or wetland to the average annual volume of the basin should be determined at this stage.

8 $$b = \frac{{{\text{V}}_{EWR} }}{{{\text{V}}_{{L_{Ann} }} or {\text{V}}_{{L _{Mon} }} }}$$

*b* = The ratio of the EWR of the lake or wetland to the average annual volume of the basin. V_*EWR*_ = Volume of environmental water requirement of the lake or wetland.

**Figure 3:**
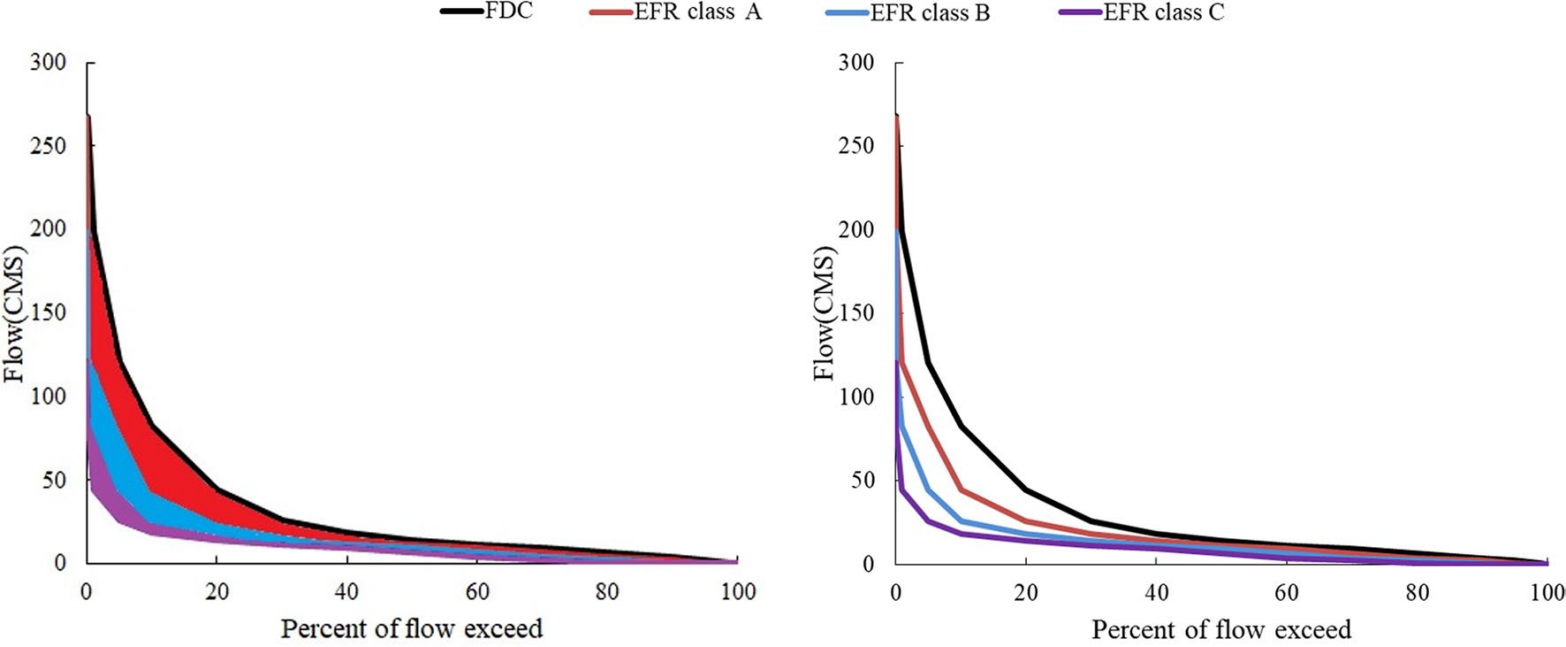
Comparison of the EFR allocated to each of the environmental management classes from this new approach (on the left) with the conventional FDCS methods (on the right).

In the conventional FDCS method, which is determined using GEFC v2.0 software^70^ (It is then called the GEFC method), depending on the type of the river EMC, the allocation curve is obtained with one or more shifts of the FDC. Each EMC includes a certain ratio of the MAF volume of the river, and changing the flow EMC facilitates changing the flow volume. It is impossible to supply a specific and predetermined downstream water volume of the river. Therefore, in the new methods, a new process must be used to calculate the amount of the FDC shift in order to provide a certain volume of water in the shifting of the FDC. First, a new definition of the EMC was developed for the new methods. In this definition, instead of using a specific shift of the FDC, the range between the two classes was characterized as an EMC. For example, the region between the curve of EMC-A and the natural flow and the region between the EMC-A and EMC-B curves are defined as EMC-A and EMC-B areas, respectively. These regions can be defined for all EMCs (Fig. 3).

Based on the new definition of the range of EMC, the FDC can be shifted as much as needed according to the volume of downstream EWR. The EWR can be defined as the annual percentage river flow respecting the shift of EMCs or a percentage between two specific classes. If the required flow volume is between two specific classes, Eq. (9) can be used to shift the FDC. In fact, with the new definition, any required probable shift can be applied to the FDC ِdiagram to reach a certain volume. In this case, new probable points are determined using Eq. (9), followed by performing the FDC shift similar to the FDCS method in the next step.

9 $$P_{{i_{new} }} = P_{i} + a{*}\left( {P_{i - 1} - P_{i} } \right)\quad i = t, \ldots ,16$$

*P*_*inew*_ = New shifted probability point. *P*_*i*_ = 17 points of FDC probability that *P*_*1*_ = 99.99% and *P*_*17*_ = 0.01%. *a* = Coefficient of shift which defined between 0 and 1. *t* = Number of shifts performed on the FDC diagram numbered 1–6 for the areas of EMC A, B, C, D, E, F, respectively.

The concept of numerical integration and Eqs. (9) and (3) were utilized to calculate the annual volume of different EMCs for each river, and Eqs. (10) and (12) were obtained for the new annual and monthly methods, respectively.

10 $$\begin{aligned} & {\text{V}}_{{AF class_{t} Ann }} = \frac{1}{{P_{1} - \left[ {P_{17} + a*\left( {P_{16} - P_{17} } \right)} \right]}} \\ & \quad \quad \quad \quad \quad *\left[ {F_{1} *\left[ {P_{1} - \left[ {P_{t + 1} + a*\left( {P_{t} - P_{t + 1} } \right)} \right]} \right] + \mathop \sum \limits_{{i = {\text{t}} + 1}}^{16} \frac{{\left( {F_{i - t} + F_{i - t + 1} } \right)}}{2}{*}\left[ {P_{i} - P_{i + 1} + a{*}\left( {P_{i - 1} - 2P_{i} + P_{i + 1} } \right)} \right]} \right]*365*24*3600 \\ \end{aligned}$$

11 $$\begin{aligned}&{\text{V}}_{{ class_{t} Mon }} = \frac{{D_{k} *24*3600}}{{P_{1} - \left[ {P_{17} + a*\left( {P_{16} - P_{17} } \right)} \right]}} \\ & \quad \quad \quad \quad \quad *\left[ {F_{1} *\left[ {P_{1} - \left[ {P_{t + 1} + a*\left( {P_{t} - P_{t + 1} } \right)} \right]} \right] + \mathop \sum \limits_{{i = {\text{t}} + 1}}^{16} \frac{{\left( {F_{i - t} + F_{i - t + 1} } \right)}}{2}{*}\left[ {P_{i} - P_{i + 1} + a{*}\left( {P_{i - 1} - 2P_{i} + P_{i + 1} } \right)} \right]} \right] \end{aligned}$$

12 $${\text{V}}_{{AF class_{t} Mon}} = \mathop \sum \limits_{K = 1}^{12} \left[ {{\text{V}}_{{ class_{t} Mon}} } \right]_{k}$$

V_*AF classt Ann*_ = AF volume for the related class of selected *t* for annual method. V_*classt Mon*_ = Monthly flow volume for the related class of selected *t* for monthly method. V_*AF classt Mon*_ = AF volume for the related class of selected *t* for monthly method.

where *t* is the number of shifts performed on the FDC diagram numbered 1–6 for the areas of EMC A, B, C, D, E, F, respectively. To find the exact value of *a* in these equations, the scope of the EMC must be determined based on the required volume by downstream. Therefore, assuming *a* = 0 in these equations, the AF volume at the boundary of each class is obtained for both EFR-Mon (Eq. (10)) and EFR-Ann (Eq. (12)) methods. The nearest calculated annual volume is selected as the appropriate EMC which is smaller than the volume of downstream. Further, the corresponding *t*-class is used to solve the equations, representing the range of the selected EMC.

At this stage, the value of the obtained ‘*a*’ from the FDC shift diagram equals the required volume of downstream. For this purpose, Eqs. (13) and (14) for the EFR-Ann and EFR-Mon methods are obtained from Eqs. (10) and (12), respectively.

13 $$b{\text{*V}}_{{AF_{Ann} }} = V_{{AF class_{t } Ann }}$$

14 $$b{\text{*V}}_{{AF_{Mon} }} = V_{{AF class_{t} Mon }}$$

By solving Eqs. (13) and (14), the obtained value of *a* represents the annual and monthly methods, and the obtained shifted FDC stands for the required annual volume downstream.

After determining the appropriate FDC, it is used to calculate the daily e-flow needs of the river using the spatial interpolation algorithm^52^, which is also employed in the FDCS method. To this end, the probability of the river flow occurrence from the annual or monthly FDCs (according to the selected method) is determined and then the required river flow in the specified probability of occurrence is obtained using the e-flow curve.

The range of variability approach (RVA)^71,72^ is a complex method based on the use of e-flow for achieving the goals of river ecosystem management. This method is applied to compare the methods and select the best one based on the least hydrological change compared to the natural flow of the river. Furthermore, it is based on the importance of the hydrological feature impact of the river on the life, biodiversity of native aquatic species, and the natural ecosystem of the river and aims to provide complete statistical characteristics of the flow regime.

In the RVA method, the indicators of hydrologic alteration (IHA) parameters related to the natural river flow are considered as a basis, and changes in the IHA parameters of different EMCs are evaluated accordingly. Richter et al.^72^ suggested that the distribution of the annual values of IHA parameters for maintaining river environmental conditions must be kept as close as possible to natural flow condition parameters. In several studies, this method was used to investigate changes in the hydrological parameters of a river over time^37^.

Moreover, the total data related to the natural flow of the river for each IHA parameter are classified into three categories in the RVA method. In this study, this classification is based on Default software, and the 17% distance from the median is introduced as the boundary of the classes. By this definition, three classes of the same size are created, in which the middle category is between 34 and 67, and the lower and higher ranges are called the lowest and highest categories, respectively.

Using the current change factor obtained from Eq. (15), the RVA method can quantify the change amount in the values of the 33 IHA parameters compared to the natural flow conditions.

15 $$HA = \left( {O_{f} - E_{f} } \right)/E_{f}$$

*HA* = Hydrological alteration index. *O*_*f*_ = Number of flows occurring within a certain category of the IHA parameter under changed flow conditions. *E*_*f*_ = Number of flows occurring in the same category specified by the parameter under natural flow conditions.

In this case, for each IHA parameter, three HA factors are obtained, which can be separately examined for river flows in these three categories. In the analysis of parameters, the positive HA means that the number of occurrences of the phenomenon has increased in a certain IHA category compared to the natural conditions of the river flow. Negative values imply a decrease in the number of occurrences of the same phenomenon. To compare the number of changes in IHA parameters, the HA factor of the RVA method and IHA software (Version 7.1)^73^ was employed to allocate e-flows in different methods. The obtained results using RVA method calculates and represents HA of each 33 parameters. However, making decision to choose the best method, all parameters need to be assessed and presented as a total index. Due to calculate total HA index based on studies of Xue et al.^74^ Eq. (16) can be used.

16 $$HA_{o} = \sqrt {\frac{{\mathop \sum \nolimits_{i = 1}^{33} HA_{i}^{2} }}{33}} *100$$

*HA*_*o*_ = Total hydrological alteration index. *HA*_*i*_ = Hydrological alteration of each of 33 parameters.

### Determination of EFR for different EMCs for all methods

Initially, the MMF for each available statistical month was obtained by daily data from stations located in the upstream of the basin rivers of Urmia Lake (Fig. 1). The FDC for the natural flow and various EMCs were obtained using MMF values and GEFC software. Next, to perform the calculations in the EFR-Ann method, the FDC of a natural flow and different EMCs during the year were plotted by daily data. Finally, for the EFR-Mon method, the daily data of each month of the year were examined and the FDC of the natural flow and EMCs were separately plotted for each month.

**Figure 4:**
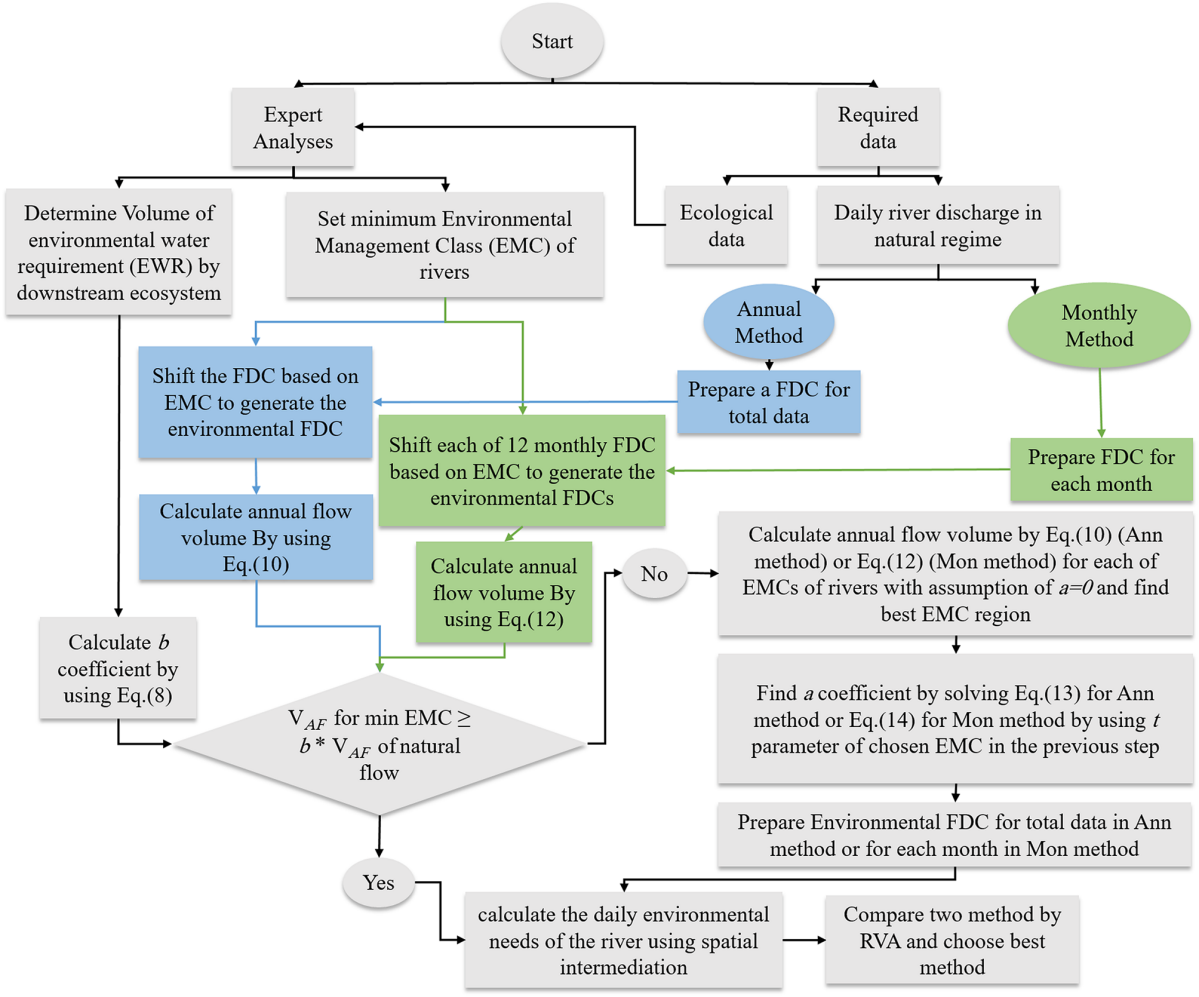
Step-by-step flowchart for determining the environmental flows of rivers in the Urmia Lake basin.

Based on the presented method in this research, Fig. 4 illustrates a step-by-step diagram for determining the e-flows of rivers in the Urmia Lake basin.

## Results and discussion

### Result of methods for different EMCs

To calculate the e-flow of the rivers entering the lake, the mean total surface water potential supplied by 10 river basins was calculated using Eqs. (6) and (7). Then, considering the volume of the EWR of the lake by the amount of 3084 Mm^3^ and using Eq. (8), the coefficient *b* was obtained to be equal to 0.705.

To select the appropriate class for e-flow allocation by Eqs. (3) and (4), the MAF volume for natural conditions and EMCs were calculated for all three methods. The percentage of AF allocation was computed by each method (Table 1).

**Table 1:** Comparison of estimated EFR from the conventional method of GEFC with two new annual (EFR Ann) and monthly (EFR Mon) methods as the percentage of the MAF of the Urmia Lake basin rivers.

River name	Station	GEFC (%MAF)			EFR ANN (%MAF)			EFR MON (%MAF)		
		Class A	Class B	Class C	Class A	Class B	Class C	Class A	Class B	Class C
Zola	Chahrigh olya	71.5	54.4	42.8	68.5	50.1	37.8	77.6	62.1	50.0
	Nazar Abad	75.9	61.9	52.1	70.9	55.4	44.1	74.8	60.0	48.6
Nazlu	Tapik	64.8	42.7	28.9	63.0	40.9	27.4	78.9	63.3	50.9
Rozeh	Kalhor	67.0	47.0	33.5	63.0	43.5	30.5	70.1	53.6	41.6
Shaharchay	Band Urmia	63.7	40.9	26.7	61.5	38.1	23.7	76.4	59.7	46.4
Barandooz	Dizaj	70.3	50.2	36.4	67.2	46.4	32.4	77.2	60.9	47.6
	Ghasemlu	48.7	31.7	24.6	48.9	29.5	19.3	54.7	36.6	26.7
Gadar	Naghadeh	65.1	41.9	27.0	60.9	37.0	22.2	77.7	61.0	47.4
Mahabad	Bitas	59.8	37.0	23.3	53.9	29.6	15.9	69.1	50.5	37.4
	Kutar	63.0	38.8	23.3	56.2	32.1	18.1	71.4	53.4	40.4
Simineh	Dashband	57.6	34.3	20.2	51.2	27.4	14.5	67.8	48.5	35.3
Zarrineh	Anian	59.0	34.7	19.6	54.0	28.7	14.6	71.2	52.6	39.3
	Panbedan	58.0	34.5	20.2	53.5	29.9	16.7	70.2	52.0	39.4
	Safakhaneh	56.9	33.2	19.7	55.3	31.3	17.9	72.8	54.7	41.2
	Senteh	59.2	34.3	19.3	54.5	29.5	15.6	71.2	52.9	39.9
Aji	Venyar	58.7	35.1	21.0	55.3	31.1	17.3	70.2	51.2	37.4
Total lake input		**61.1**	**38.2**	**24.0**	**57.1**	**33.6**	**19.9**	**72.5**	**54.7**	**41.6**

According to Table 1, the ratio of allocated e-flow to the total river flow is not necessarily identical for different rivers in the same classes. The nature of the river and its FDC shape can differ so that the maximum and the minimum flow percentages are related to Tapik and Ghasemlu stations in the EFR-Mon method, respectively. The comparison of the flow ratio values of an EMC in each of the three proposed methods revealed that the allocation percentage of the conventional GEFC method, in terms of the annual allocation volume, is between the EFR-Ann and EFR-Mon methods in all rivers. As mentioned in section “Ordinary method of flow duration curve shifting (FDCS) in estimating e-flow” of this research, based on previous studies, GEFC class C method is considered as the minimum EMC for rivers in Lake Urmia basin. However, according to Table 1, if the GEFC class C method is applied, the volume of water allocated to the lake will be equal to 24.0% of the AF volume. Although GEFC class C method has been selected as an acceptable class for rivers of Lake Urmia basin in the past, but the application of this method will gradually make the situation of the lake more critical. Considering the volume of e-flow of higher EMCs of GEFC method in Table 1, the maximum volume of flow that can be allocated to the lake in GEFC class A is 61.1%. Due to the lake's need for 70.5% of the annual surface water volume of all studied rivers, this method will not be able to provide the required flow of the lake in conventional EMCs. Among the two new proposed methods, EFR Ann class A can allocate a maximum of 57.1% of the total annual flow of rivers to the lake, which is not enough to rehabilitate the lake and achieve ecological level. According to Table 1, it can be concluded that among the EMCs of the presented methods, only EFR Mon class A with 72.5% AF volume will be able to supply the required water to the lake. The use of conventional EMCs caused that the methods used did not have enough flexibility and the volume of water required by the lake was not provided properly with acceptable accuracy. For example, in the annual method, none of the offered EMCs will be able to supply water to the lake and will make it impossible to use this method. Also, if EFR Mon class A is used, the volume of water allocated to the lake will be 72.5%, which is 2.0% higher than the required amount of the lake. This will limit the methods that can be used and impose more or less flow allocation than the EWR. Therefore, to overcome these limitations, the class ADJ presented in this research has been used.

### New adjusted method to allocate EWR of the lake

Based on the coefficient b = 0.705, 70.5% of the AF volume of each of the 10 main rivers of the basin is released to meet the EWR of the Urmia Lake. Data in Table 1 indicate that none of the rivers in any of the EMCs have an exact amount of 70.5% of the AF. To achieve this amount, it is necessary to use the facilities of the newly proposed methods. Therefore, the appropriate value of coefficient “*a*” in the EFR-Ann and EFR-Mon methods is calculated by solving Eqs. (13) and (14). To this end, first, it is necessary to obtain factor *t* in Eqs. 10 and 11, which represent the range of the selected EMC. For this purpose, data in Table 1 are used and the EMC is selected for each river according to the findings of Table 2 in which 70.5% of the flow is located, Followed by obtaining the corresponding *t*-factor. For example, in Table 1, the amount of 70.5% of the AF required by the lake for Tapik Nazlu station is higher than EFR Ann class A with 68.5% of the AF. According to the new definition of the range of EMCs shown in Fig. 3, the shifted curve will be located in the range of class A and therefore the value of *t* will be equal to 1. While in the monthly method the amount of 70.5% of the flow will be in the range between class A and B, which according to the new definition of EMCs (Fig. 3), this range will be in class B and the value of *t* will be equal to 2. Similarly, for all rivers, the occurrence zone of allocating 70.5% of the AF volume has been determined, and the corresponding *t* was selected, which can be seen in Table 2.

**Table 2:** Selection of EMCs and corresponding t-values for two new annual (EFR Ann) and monthly (EFR Mon) methods based on providing 70.5% of the annual flow of each of the rivers as the EWR.

River name	Station	EFR Ann		EFR Mon	
		Class	t	Class	t
Zola	Chahrigh olya	A	1	B	2
	Nazar Abad	B	2	B	2
Nazlu	Tapik	A	1	B	2
Rozeh	Kalhor	A	1	A	1
Shaharchay	Band Urmia	A	1	B	2
Barandooz	Dizaj	A	1	B	2
	Ghasemlu	A	1	A	1
Gadar	Naghadeh	A	1	B	2
Mahabad	Bitas	A	1	A	1
	Kutar	A	1	B	2
Simineh	Dashband	A	1	A	1
Zarrineh	Anian	A	1	B	2
	Panbedan	A	1	A	1
	Safakhaneh	A	1	B	2
	Senteh	A	1	B	2
Aji	Venyar	A	1	A	1

According to the results ofTable 2, the annual method, the major EMC that is selected to supply 70.5% of AF volume is class A. While in the monthly method, the major selected EMC for supply this amount of water is class B.

### Comparison of the methods

Although Urmia Lake has 10 main rivers, only the Nazlu River was discussed in this study due to the similarity of the results in the study of rivers leading to the lake.

**Figure 5:**
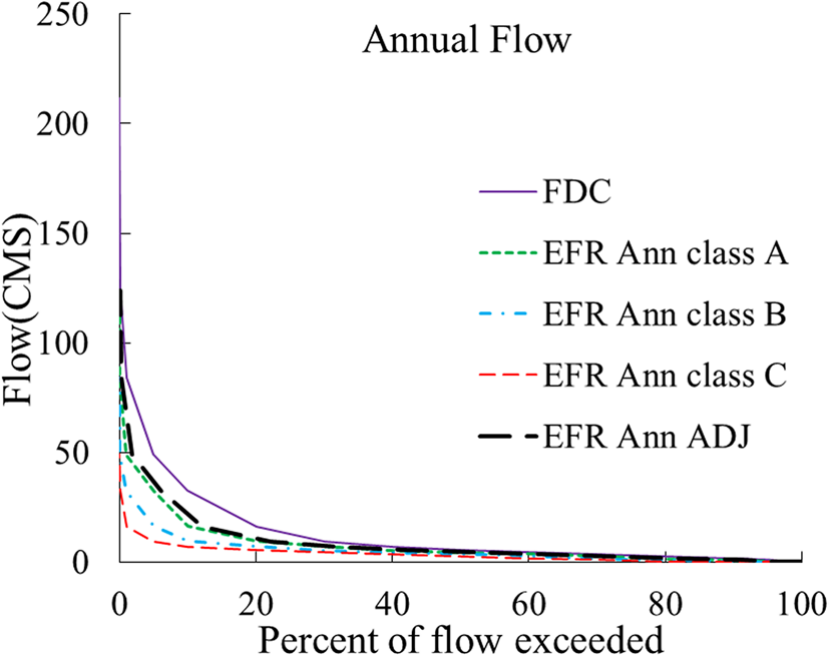
Comparison of annual FDCs for different EMCs with the shifted FDC based on the adjusted EFR of the lake at the Tapik Station, Nazlu River.

**Figure 6:**
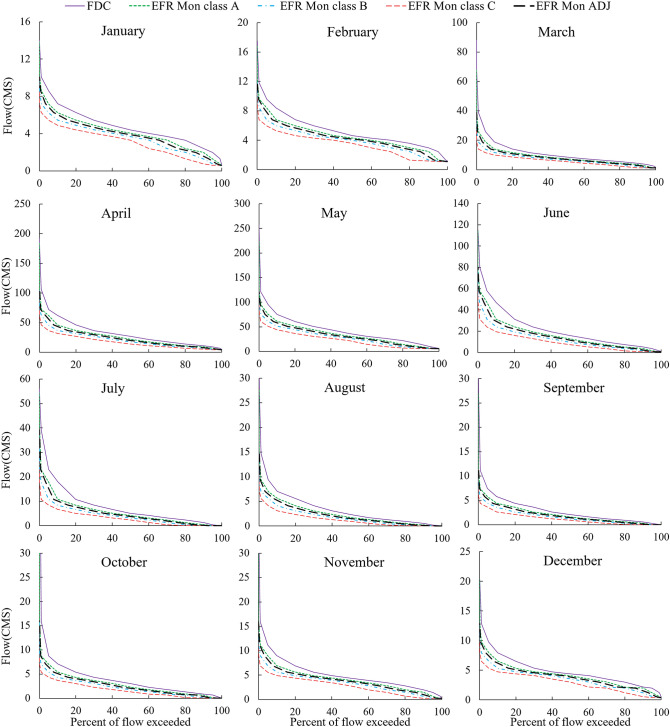
Comparison of monthly FDCs for different EMCs with the shifted FDC relying on the adjusted EFR of the lake at the Tapik Station, Nazlu River.

Using the obtained *t*-values (Table 2.), Eqs. (13) and (14) are solved for each of the rivers, and a new shifted FDC diagram is obtained for passing 70.5% of the annual river flow for both new methods. The FDC for estimating the EFR- Ann—ADJ and EFR- Mon—ADJ environmental needs are demonstrated according to the lake EWR in comparison with EMCs A, B, and C in Figs. 5 and 6, respectively.

**Figure 7:**
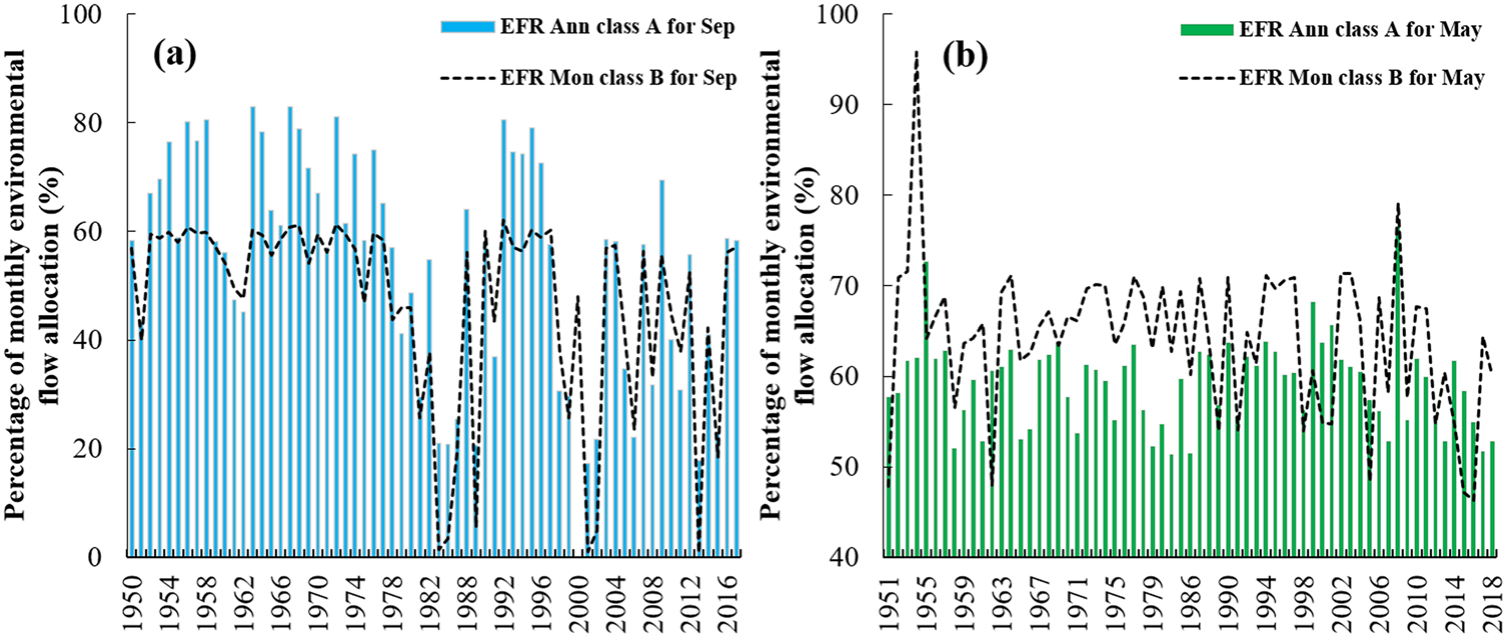
Percentage of monthly environmental flow allocation relative to the natural flow capacity with the comparison of the EMC-A of the annual method with the EMC-B of the monthly method at the Tapik Station, Nazlu River: (**a**) September and (**b**) May.

Due to the difference in the probability of the occurrence of a specific flow during different months of the year, a simple FDC cannot be used as a flow characteristic of the river during the whole year. For example, a flow of 10 CMS at the Tapik Station on the upstream of Nazlu River in July is considered to be a high flow with a 20% probability rate while it is considered to be a low flow with a 95% probability of occurrence in May when the river has the highest MMF of the year (Fig. 6). However, in the EFR-Ann method, the probability of the flow of 10 CMS is 31% and the allocation of e-flow is based on the probability of flow occurrence regardless of the month of occurrence. Therefore, the nature of the hydrological change of the river can be used to allocate the e-flow in different months of the year by the monthly method. To prove this issue, Fig. 7 displays the ratio of the e-flow allocation volume relative to natural flow conditions in July and May at the Tapik Station on the Nazlu River for the EMC-A of annual method and the EMC-B of the monthly method. Based on calculations, both of these EMCs provide 63% of the annual river flow volume as downstream EWR during the year.

According to Fig. 7, when monthly FDC is higher than the annual, the allocated flow volume to the downstream of the river in the EFR-Mon method in high flow months will be more compared to the EFR-Ann method. During the statistical period of Tapik Station for May, the EFR-Mon method allocates an average of 14% more flow volume to the downstream of the river in comparison with the EFR-Ann method. Examining the data of this station, it was found that the EFR-Mon method allocates smaller amounts of flow in low flow months when the monthly FDC is lower than the annual compared to the annual to downstream. Therefore, the EFR-Mon method allocates 21% less amount of water to the downstream in September compared to the EFR-Ann method. According to the analysis, this difference between the EFR-Ann and EFR-Mon methods annually increases with a decrease in the percentage of the allocated flow volume to the downstream compared to the natural flow regime.

To supply the volume of the EWR of the Urmia Lake from the Tapik Station of Nazlu River, the shifted FDC is located in the EMC-A region according to Fig. 5 by the annual method (EFR-Ann-ADJ) while the shifted FDC is located in the EMC-B region in the monthly method (EFR-Mon-ADJ) according to Fig. 6. To determine the average volume of the allocated flow using the EFR-Mon method in different months of the year, the volume of each EMC and the class-ADJ are calculated using Eq. (11) and related data are presented in Table 3.

**Table 3:** Monthly distribution of EFR values as the percentage of the MMF using the new monthly method (EFR Mon) in different conventional EMCs and the adjusted class of river management at the Tapik Station, Nazlu River.

Month	EFR Mon (%MMF)			
	Class A	Class B	Class C	ADJ
January	86.3	74.3	63.5	79.9
February	86.2	74.4	64.0	79.8
March	79.1	64.3	52.9	71.1
April	79.4	64.4	52.3	71.3
May	80.8	65.9	53.7	72.7
June	75.7	57.6	43.7	65.9
July	71.0	51.3	37.2	60.4
August	72.3	52.8	38.3	61.7
September	73.5	54.9	40.5	63.4
October	71.3	54.6	41.9	62.0
November	79.9	65.0	52.8	71.8
December	83.7	70.6	59.3	76.6
Total (%MAF)	78.9	63.3	50.9	70.5

Table 3 indicates the ratio of e-flow allocation of each month to the average natural flow of that month. According to Table 3, the river shows a unique behavior in each month of the year and allocates different amounts of flow as e-flow for different EMCs. Data in Table 3 indicate that, the highest ratio for all four EMCs is in January and February, and the lowest is in July. The maximum difference between the values of the flow allocation ratio among different months in EMCs A, B and C is 15.3, 23.1 and 26.8, respectively. It can therefore be concluded that this difference increases with decreasing EMCs.

### Methods evaluation by RVA

**Figure 8:**
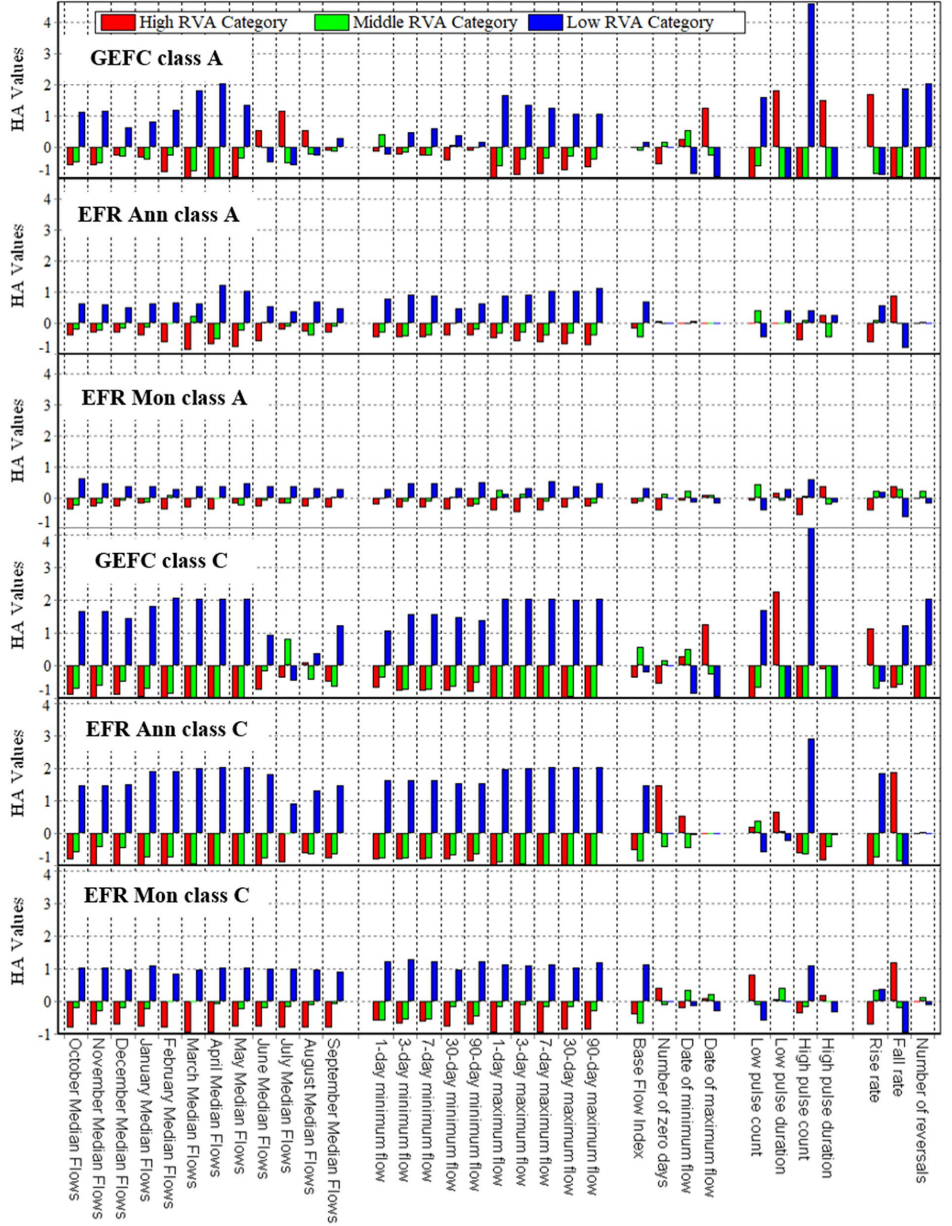
HA values using the RVA method for EFR allocations in two A and C EMCs with GEFC, annual, and monthly approaches at the Tapik Station, Nazlu River.

The RVA method was used to better evaluate the number of changes and to compare the new methods presented with the conventional FDCS method (GEFC), and the diagram of hydrological alterations is depicted in Fig. 8.

**Figure 9:**
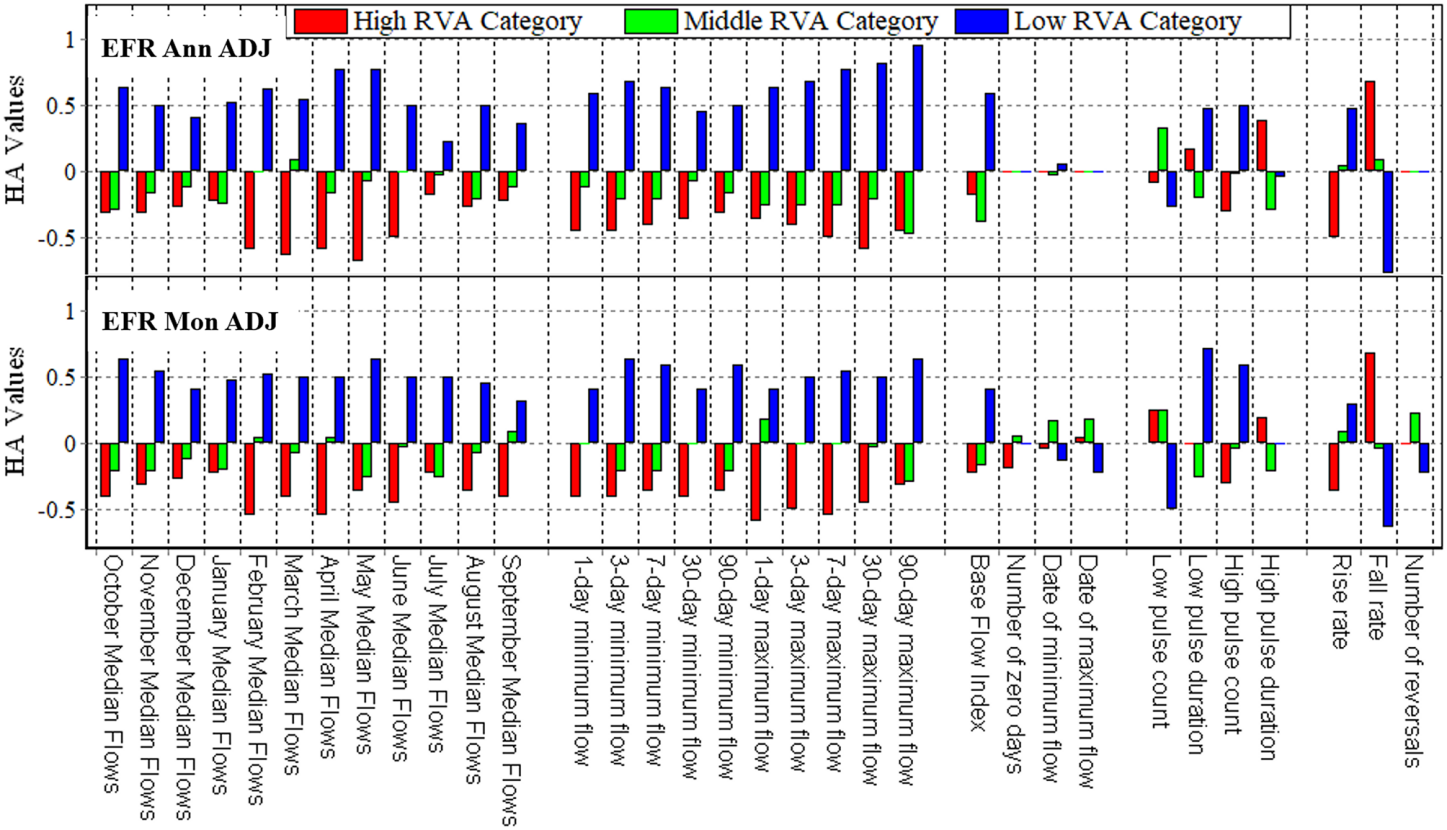
HA values using the RVA method for EFR allocations using adjusted annual and monthly methods at the Tapik Station, Nazlu River.

Figure 8 illustrates the HA of parameters for the two new EFR-Ann and EFR-Mon methods and the conventional method (GEFC) for classes A and C regarding examining the trend and magnitude of these changes. According to this diagram, the HA parameters of the GEFC method are extremely higher than those of the newly presented methods. Using absolute averaging (regardless of the negative or positive values of the changes), HA for GEFC class A in the range high, middle, and low RVA categories are 0.60, 0.38, and 0.85, respectively. However, the absolute average value of HA for the mentioned categories is 0.35, 0.18, and 0.51, as well as 0.23, 0.12, and 0.28 in the EFR Ann class A and EFR Mon class A methods, respectively. Based on data in the diagram in Fig. 8, selecting a lower EMC increases the HA in hydrological parameters and the EFR-Mon method provides better results. To prove this point, using the absolute averaging of HA for the GEFC class C method, the RVA categories were examined in the high, middle, and low ranges, which were 0.68, 0.59, and 1.20, respectively. However, the absolute average value of HA for the mentioned categories was 0.69, 0.52, and 1.12, as well as 0.54, 0.20, and 0.69 in the EFR Ann class C and EFR Mon class C methods, respectively. According to the presented results, both new methods provide better results compared to the GEFC method and bring less HA to the river ecosystem. However, to compare the results of EFR-Ann and EFR-Mon methods of supplying the EWR of the lake, a diagram of HA was used for the class-ADJ in both new methods, which supplies the Urmia Lake. In the class-ADJ, the FDC has shifted in such a way that both methods transferred 70.45% of the MAF volume to the lake (Fig. 9).

According to Fig. 9, the absolute mean HA values for the high, middle, and low RVA classification intervals for the EFR Ann ADJ method are 0.28, 0.13, and 0.40, respectively. However, the absolute average of HA for the EFR Mon ADJ method is 0.27, 0.11, and 0.36. Additionally, the standard deviation (SD) of the absolute HA values of both methods was calculated for three RVA classes, and the SD value for the high, middle, and low classes was 0.22, 0.13, and 0.30, as well as 0.20, 0.10, and 0.24 in the EFR Ann ADJ and EFR Mon ADJ methods, respectively. Based on the obtained data (Fig. 9) and the calculated SD, HA has a more uniform trend for the monthly method compared to the annual method in different parameters.

To determine the amount of hydrological changes for each method using Eq. (16), the amount of HA_o_ which is the best representor of hydrological changes of the river is calculated considering all 33 parameters. The results are illustrated in Table 4.

**Table 4:** Comparison of HA_o_ value of conventional GEFC method with two new methods annual (EFR Ann) and monthly (EFR Mon) for different EMCs.

Method	HA_0_			Average of three Categories (%)	%MAF
	Middle RVA Category (%)	High RVA Category (%)	Low RVA Category (%)		
EFR Ann ADJ	20.2	39.9	55.3	38.5	70.5
EFR Ann class A	27.6	48.8	69.3	48.5	63.0
EFR Ann class C	71.7	91.7	158.4	107.3	27.4
EFR Mon ADJ	16.6	37.8	48.5	34.3	70.5
EFR Mon class A	17.2	31.1	37.8	28.7	78.9
EFR Mon class C	30.0	73.3	94.2	65.8	50.9
GEFC class A	56.9	86.5	134.9	92.8	64.8
GEFC class C	77.2	92.7	169.2	113.0	28.9

Regarding Table 4 average of HA_o_ values for GEFC method are higher than presented method. Also, GEFC class A method allocates 64.8 percentage of MAF to e-flow and the average of HAo of the river is 92.8 percent. However, EFR Ann class A and EFR Mon class C allocates 63.0 and 50.9 percent of MAF, respectively. Both of classes allocate lower amount of water as e-flow, although, they contain lower HA_o_ than GEFC method. This is because of the way of flow allocation in GEFC method which causes severe hydrological changes rather than two new Mon an Ann methods. Finally, based on the results of the Table 4, EFR Mon class A and EFR Mon ADJ have first and second place in applying the least hydrological changes, respectively. The allocated water amount of EFR Mon class A is 8.4 percent (MAF%) higher than the EFR Mon ADJ, so this causes lower HA_o_. The EFR Mon ADJ is capable of supplying the required water of the lake and this result makes it the best method to supply the rivers EFR of the basin.

### The impact of deferent methods on allocating water to the lake

**Figure 10:**
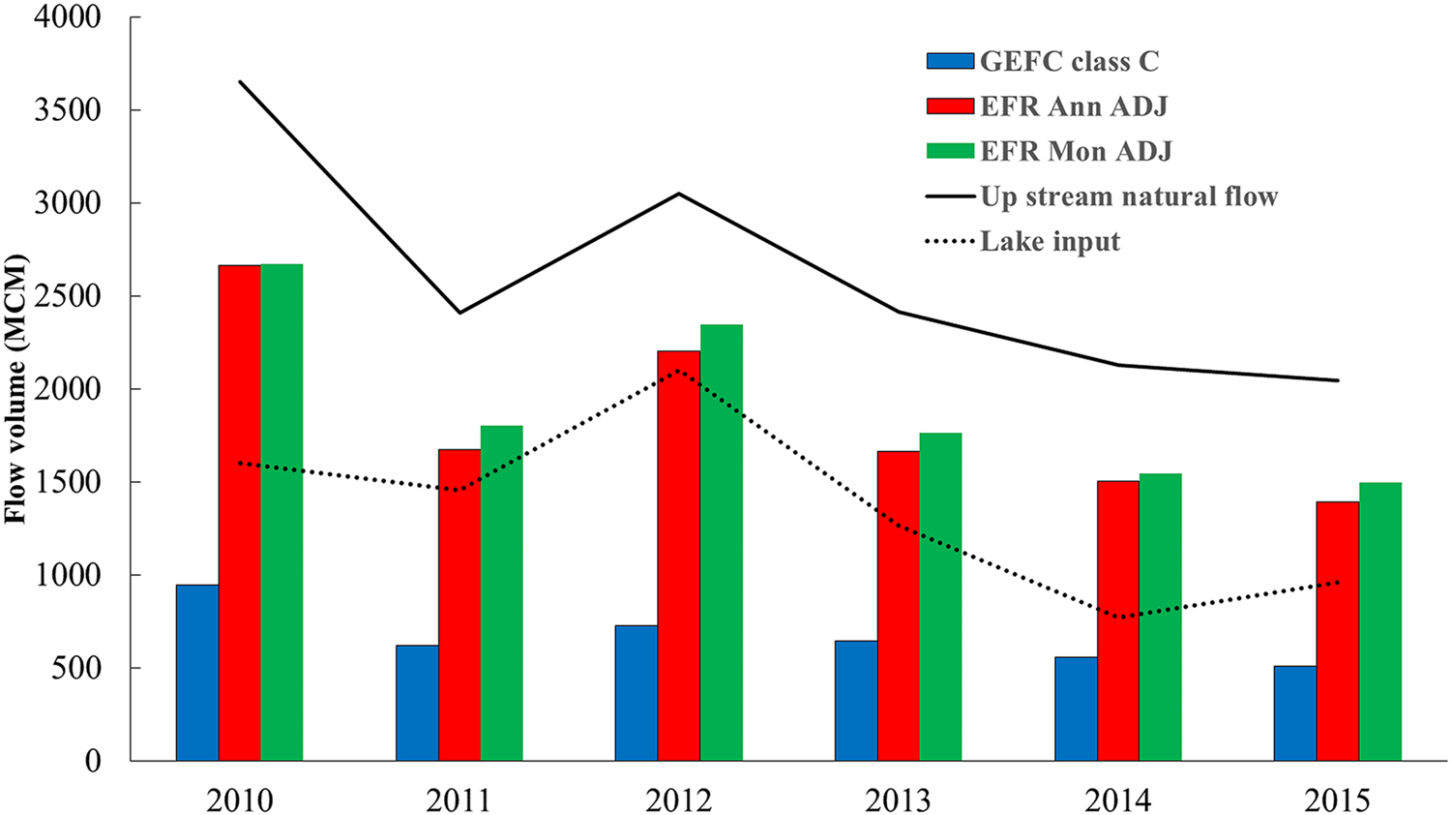
Comparison of environmental flow allocation from two new annual and monthly methods with the conventional GEFC method and with natural river flow capacity and water entering from 10 main rivers to the lake in 2010–2015.

After calculating the EFR of the inflow rivers to the Urmia Lake for both new methods, the total volume of water allocated to the 10 main inflow rivers to the lake (2010–2015) was examined for each method (Fig. 10).

Based on the results (Fig. 10), the GEFC class C has the lowest volume of allocated water concerning the EWR of the lake (3084 Mm^3^) in all six years, and on average, 26% of the annual volume of the natural river flow equivalent to 670 Mm^3^ of water is delivered to the lake each year. Further, the average volume of the allocated water to the Urmia Lake in the EFR Ann ADJ with 71% and the EFR Mon ADJ with 74% of the natural flow of the river will be equivalent to 1852 and 1938 Mm^3^, respectively. The average natural flow volume of rivers was equal to 2618 Mm^3^ during 2010–2015, which equals 60% of the long-term average natural flow volume of these rivers.

According to Fig. 10, the allocated flow to the Urmia Lake in these six years is extremely higher than the calculated volume through the GEFC class C method, but only 44% of the EWR of Urmia Lake has been supplied by the 1361 Mm^3^ allocation of water to it. However, if the EWR of the Urmia Lake is considered by Eq. (8), the water volume equal to or more than 70.5% of the natural flow of the river as the e-flow should be allocated to the lake. Accordingly, the volume of water that can be allocated to Urmia Lake must be more than 1844 Mm^3^, which is equal to 60% of the lake EWR, implying that both EFR Ann ADJ and EFR Mon ADJ methods can supply this amount of water.

## Conclusion

These new methods are designed to bridge the hydrological method of FDCS, which provides the required e-flow of the river and the EWR supply for the wetland and lake downstream of the rivers. Using the proposed methods, the river flow can provide the EWR in which they make the least changes to the natural flow regime. Two new methods (EFR-Ann and EFR-Mon) were introduced in this research. Both methods could create a connection between the lake and the river. Based on the results, the EFR-Mon method makes the least HA (based on studies using the RVA method) due to considering the hydrological conditions of the flow during different months of the year. Therefore, it is the most appropriate method in estimating the e-flow of rivers leading to wetlands and lakes by considering the EWR for these aquatic ecosystems compared to the EFR-Ann method. Compared with the conventional method that can be calculated through GEFC software, using daily data and performing e-flow calculations in real-time are among the most important advantages of the two new methods, which can calculate the EFR value on each day of the year according to the upstream inflow and allocate it to the downstream. However, using the conventional FDCS method, due to the use of average monthly data, it is impossible to check the adequacy of the allocated e-flow before obtaining the average monthly flow at the end of each month. Based on the studies using the RVA method in this research, the monthly method was the most appropriate alternative in estimating the e-flow of rivers compared to the annual method due to considering the hydrological conditions of the flow during different months of the year and making the least hydrological change. In addition, in the long run, both new methods allocate EWR to Urmia Lake and restore it by allocating 70.5% of the AF volume of the basin rivers. The use of the new methods have reduced the impact of expertise biases^75^ and thus increased the reliability of these methods by creating a suitable quantitative criterion to determine the shifting amount of the FDC and allocate the appropriate flow volume to provide the EWR of downstream lakes and wetlands.

## Supplementary Information

Supplementary Figures.
