# Supplementary material for: New integrated hydrologic approach for the assessment of rivers environmental flows into the Urmia Lake
Source: Sci Rep. 2022 May 16;12:8110. doi: 10.1038/s41598-022-10262-4 (PMC9110391; doi:10.1038/s41598-022-10262-4)
Supplement: Supplementary file 1 — Supplementary Figures. [file 41598_2022_10262_MOESM1_ESM.docx]

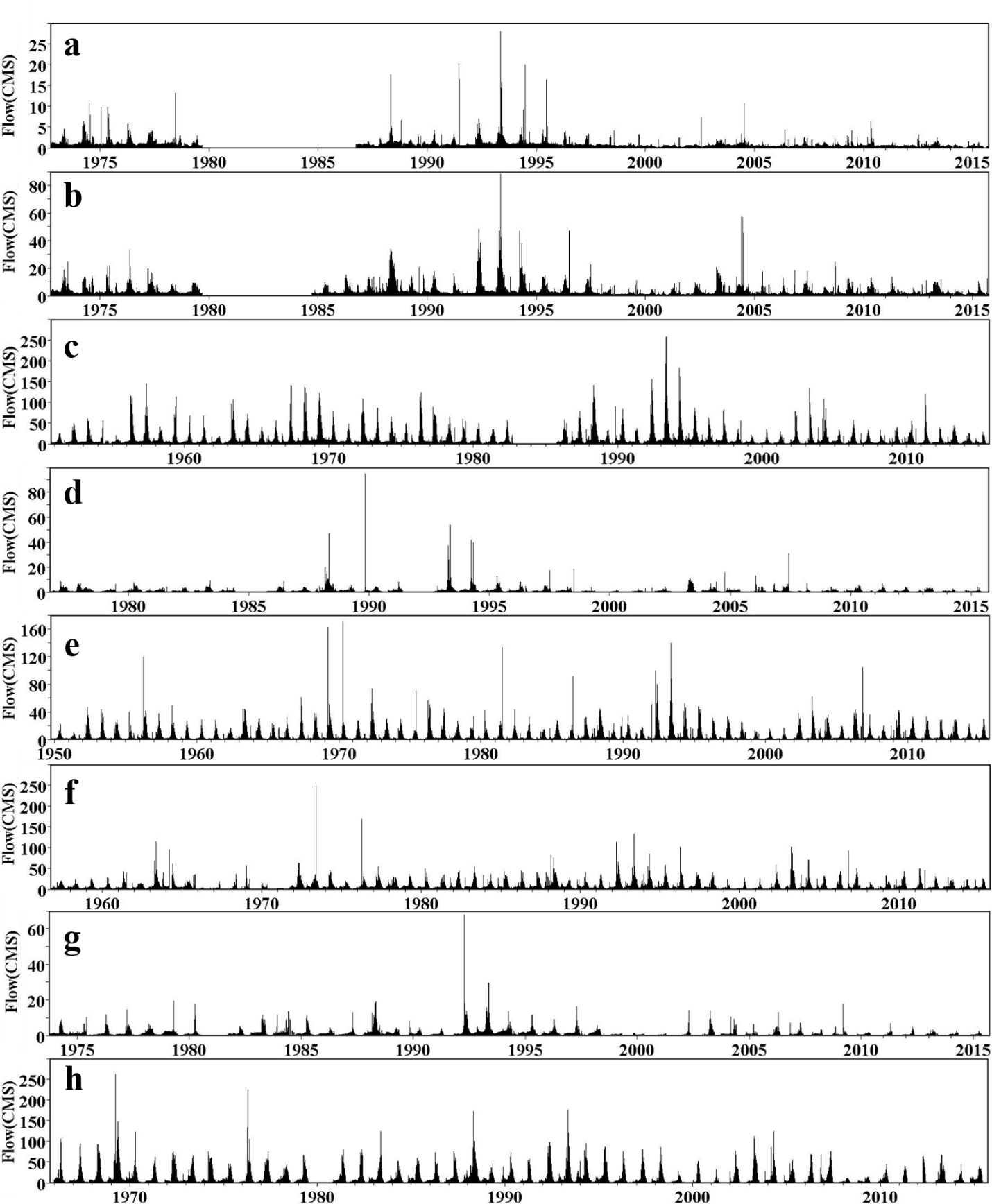


Figure S1- Historical hydrograph of studied rivers. a) Nazarabad Station, Zola River; b) Chahrigholya Station, Zola River; c) Tapik Station, Nazlu River; d) Kalhor Station, Rozeh River; e) Band Urmia Station, Shahar River (naturalized hydrograph); f) Dizaj Station, Barandouz River; g) Ghasemlu Station, Barandouz River; h) Naghadeh Station, Gadar River.
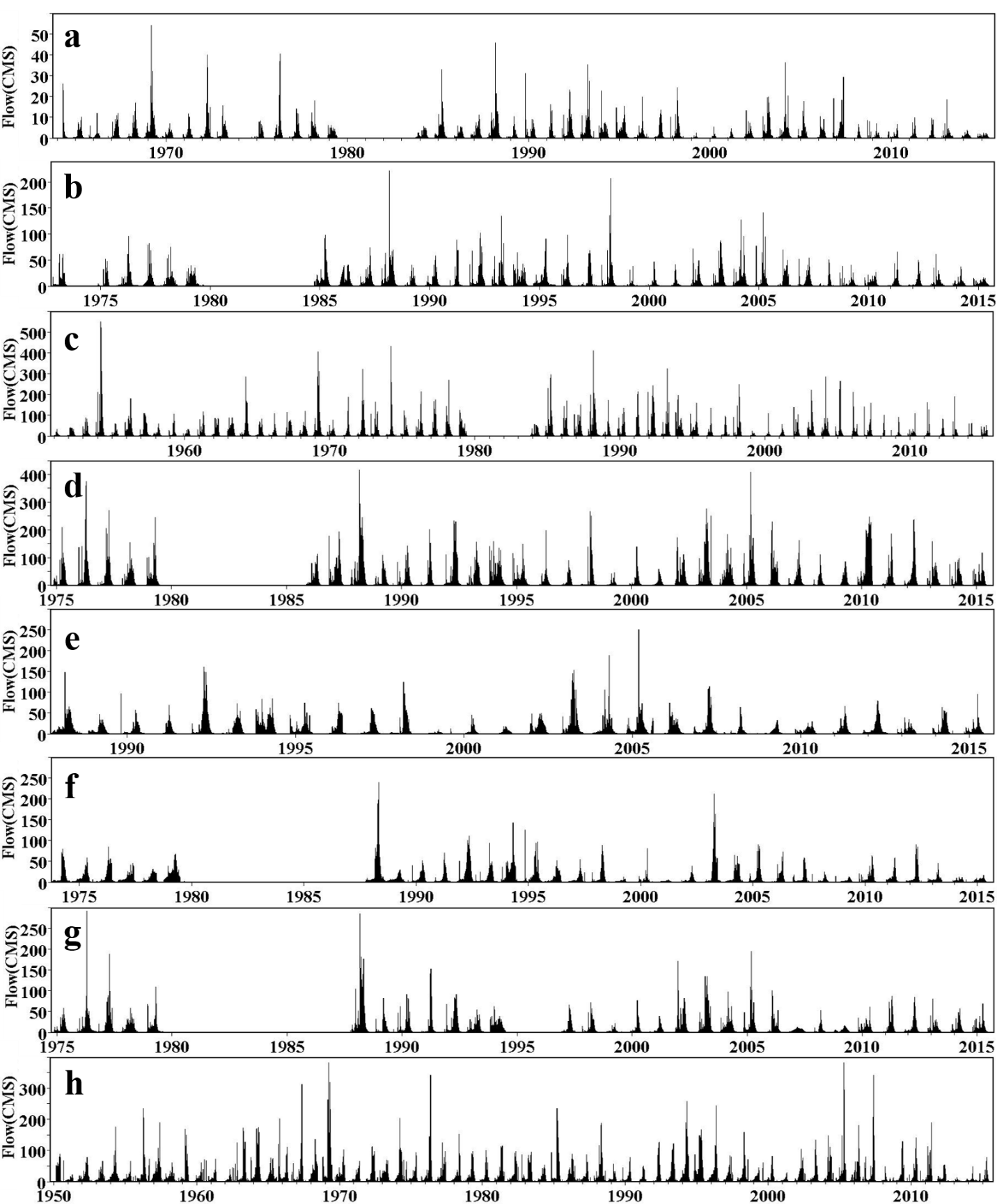


Figure S2-Historical hydrograph of studied rivers. a) Bitas Station, Mahabad River; b) Kutar Station, Mahabad River; c) Dashband Station, Simineh River; d) Anian Station, Zarineh River; e) Senteh Station, Zarineh River; f) Safakhaneh Station, Zarineh River; g) Panbedan Station, Zarineh River; h) Venyar Station, Aji River (naturalized hydrograph).
